# Resonant Effects in Nanoscale Bowtie Apertures

**DOI:** 10.1038/srep27254

**Published:** 2016-06-02

**Authors:** Li Ding, Jin Qin, Songpo Guo, Tao Liu, Edward Kinzel, Liang Wang

**Affiliations:** 1 Department of Optics and Optical Engineering, Anhui Key Laboratory of Optoelectronic Science and Technology, University of Science and Technology of China, Hefei, Anhui 230026, China; 2 Mechanical and Aerospace Engineering, Missouri University of Science and Technology, Rolla, MO, 65409, USA

## Abstract

Nanoscale bowtie aperture antennas can be used to focus light well below the diffraction limit with extremely high transmission efficiencies. This paper studies the spectral dependence of the transmission through nanoscale bowtie apertures defined in a silver film. A realistic bowtie aperture is numerically modeled using the Finite Difference Time Domain (FDTD) method. Results show that the transmission spectrum is dominated by Fabry-Pérot (F-P) waveguide modes and plasmonic modes. The F-P resonance is sensitive to the thickness of the film and the plasmonic resonant mode is closely related to the gap distance of the bowtie aperture. Both characteristics significantly affect the transmission spectrum. To verify these numerical results, bowtie apertures are FIB milled in a silver film. Experimental transmission measurements agree with simulation data. Based on this result, nanoscale bowtie apertures can be optimized to realize deep sub-wavelength confinement with high transmission efficiency with applications to nanolithography, data storage, and bio-chemical sensing.

Nanoscale ridge apertures defined in metallic films can be used to focus electrometric fields well below the diffraction limit^1^,^2^,^3^,^4^. This has generated significant theoretical and experimental interest^5^,^6^,^7^,^8^,^9^,^10^,^11^,^12^,^13^,^14^,^15^. The intense confined near-field allows nanoscale ridge apertures to be used for nanolithography^16^,^17^, data storage^18^, bio-chemical sensing^19^ and many other areas where high optical resolution and field enhancement are critical. The bowtie aperture is one type of ridge aperture. It consists of two open arms separated by ridges which define a nanometer-sized gap^20^. When the aperture is illuminated by a light source polarized across the gap (electric-field parallel to the ridges), the open arms provide longer cutoff wavelengths while the confinement of the spot is governed by the gap size. The longer cutoff wavelength provides orders of magnitude larger transmission than circular aperture with the same level of sub-wavelength electric field confinement^21^,^22^. The transmission through the aperture exhibits a significant spectral dependence, with multiple transmission peaks. At resonance the far-field transmission and near-field intensity can be more than an order of magnitude greater than the (non-cutoff) off-resonant aperture. Understanding the nature of this resonance is critical for optimizing these apertures in practical applications. Despite this significance, the origin and nature of the bowtie aperture spectral resonance has not been fully resolved.

In this paper, the transmission spectral resonance is studied numerically for different sized bowtie apertures in different silver film thicknesses. We show that the spectral response of the bowtie aperture is dominated by Fabry-Pérot resonance and plasmonic resonance. While the dominant modes for the aperture are similar to the waveguide mode, this becomes hybridized with long-range plasmonic modes. This effect is dramatic as the frequency approaches the plasma frequency of the metal. This dramatically affects the transmission spectra. To verify the numerical results, isolated bowtie apertures are focus ion beam milled in a silver film. The far-field transmission through the isolated bowtie apertures is measured and shown to agree with numerical predictions. A correct understanding of the bowtie waveguide modes significantly affects aperture design and performance.

## Results and Discussion

**Figure 1:**
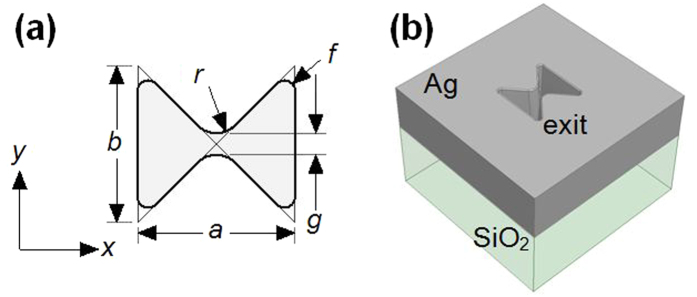
Bowtie aperture geometry (**a**) dimensions and (**b**) 3D rendering of aperture.

Ridge waveguides are formed by loading a regularly shaped waveguide with one or more conducting ridges. This has the benefit of increasing the cutoff wavelength of the lowest mode Transverse Electric (TE) mode of the waveguide and increases the separation between this mode and higher-order modes. The electric field is confined to the gap between the ridges which may significantly smaller than the free space wavelength of the radiation, while the open arms of the waveguide allow the magnetic field to circulate. At optical frequencies, this sub-diffraction limited focusing is of considerable interest because the field confinement is limited by the ability to define nanoscale gaps in metal films. While sharp edges at the ridges further concentrate the electric field, these are difficult to fabricate at nanoscale length scales. Because the performance of the aperture is strongly dependent on its geometry it is necessary to define it realistically. Figure 1 shows the geometry of the apertures studied in this paper. The aperture consists of a short section of rectangular waveguide defined by length, *a*, and width *b*. The waveguide is loaded with two conducting triangular ridges whose apexes meet center of the aperture (initially no gap). A radius *r* is applied to both ridges, to define a gap, *g*, defined by:


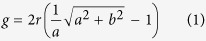


Finally, the four exterior corners are filleted with a radius *f*. This modified geometry is more realistic than previous parametric models^19^,^20^ whose sharp corners produce non-physical field concentrations. *r* = 30 nm and *f* = 10 nm are used for the simulations presented in this paper unless otherwise noted. These dimensions are selected based on observations of focus-ion beam milled apertures and correspond to a gap of 25 nm for an aperture defined by *a* = *b* = 200 nm.

**Figure 2:**
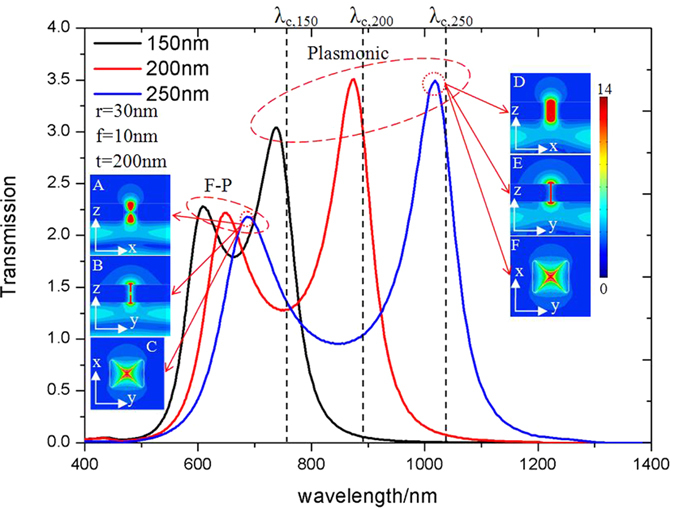
Transmission efficiency through different sized bowtie apertures defined in a *t* = 200 nm thick silver film, black line: *a* = *b* = 150 nm; red line: *a* = *b* = 200 nm; blue line: *a* = *b* = 250 nm; *λ*_*c*,150_, *λ*_*c*,200_, and *λ*_*c,*250_ denote the cutoff wavelengths of the corresponding bowtie waveguides; insets (A)–(F) show the electric fields distributions at two resonant peaks for the *a* = *b* = 250 nm aperture.

Figure 2 shows the simulated transmission spectrum of different sized (*a* = *b*) bowtie apertures defined in a 200 nm thick silver film on top of a fused silica substrate. Two distinct peaks corresponding to resonant modes are observed in the transmission. The peak transmission efficiency (normalized to the open area of the aperture) exceeds unity which is consistent with a resonant antenna while the peak intensity at the exit is more than 14x larger than the incident beam. The resonant wavelength scales nearly linearly with the size of the aperture while ratio between the transmission peaks varies with the size of the aperture and is significantly lowers for smaller apertures. These peaks correspond to different longitudinal modes in the aperture. This is apparent from the field distribution which is illustrated in insets of Fig. 2(A,B,D,E). Figure 2C,F show that the transverse field intensity distribution at the exit of the aperture does not depend on the longitudinal mode due to the fact that they share a common waveguide mode.

**Figure 3:**
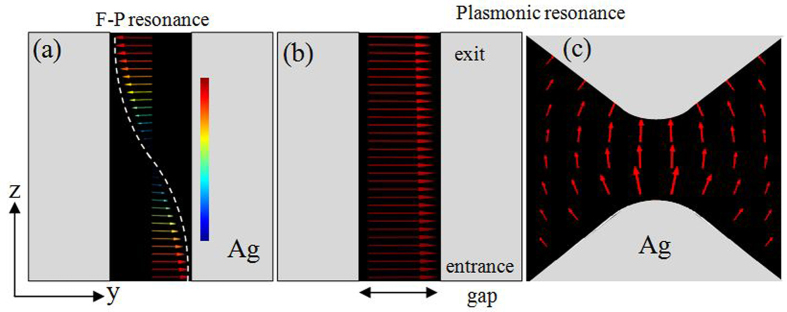
Vector electric field distributions in *yz* plane for bowtie aperture defined by *a* = *b* = 250 nm and *t* = 200 nm, (**a**) *yz* plane for 1st order F-P resonance wavelength, λ = 690 nm and (**b**) *yz* plane for 0^th^ order F-P resonance, λ = 1028 nm. (**c**) Vector electric field distributions on exit xy plane at λ = 1028 with the gap geometry is an enlarged to show the field distribution.

The peak vector electric field distributions of the *a* = *b* = 250 nm bowtie aperture is shown in Fig. 3. At *λ*_0_ = 690 nm, the peak field intensity occurs at the entrance and exit of the bowtie aperture with a minimum midway through the aperture. Standing waves are visible in the vector field distribution and correspond to longitudinal Fabry-Pérot modes. In contrast to the F-P resonance, for the *λ*_0_ = 1028 nm peak, the electric field shows minimal variation along the length on the aperture. At the entrance and exit of the aperture, electric charge accumulates at the ridges. The dipole like charge distribution results in strong electric field enhancement and is similar to the fundamental plasmonic mode of resonant nanoparticles^23^.

The silver film supports Surface Plasmon Polaritons (SPP) and the excitation of the bowtie aperture generates an oscillation of conduction electrons in the conductive ridge. The SPP wavelength is proportional to the effective oscillation path length of the conduction electrons^24^. This hybridizes with the transverse TE mode of the bowtie aperture and serves to decrease its cutoff wavelength over what it would be in a perfect conduction. The -P resonance is slightly blue-shifted from the cutoff wavelength, which scales as the size of the aperture increases.

**Figure 4:**
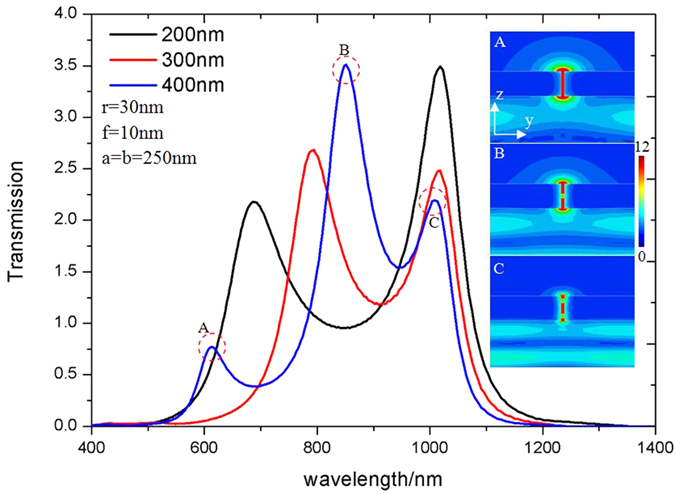
Transmission efficiency through the bowtie apertures with *a* = *b* = 250 nm defined in silver films of different thicknesses, black line: *t* = 200 nm; red line: *t* = 300 nm; blue line: *t* = 400 nm; with insets (A–C) showing the electric field distribution in yz plane at the three resonant peaks for the aperture defined in a *t* = 400 nm film.

The nature of the modes is further illustrated in Fig. 4 which shows the transmission spectra for *a* = *b* = 250 nm apertures defined in different thickness silver films. As the thickness of the bowtie aperture increases, the F-P resonance wavelength is red-shifted. This is due to the fact that the Fabry-Pérot cavity resonance is determined by *k*_z·_*t* = *m*·π, where *m* is an integer and *k*_*z*_ is the propagation constant, 2π*n*_*eff*_/λ_0_. However, the plasmonic resonance remains the same when the thickness of the silver film changes. This film thickness independence of the resonant wavelength is consistent with the homogeneous vector field distributions of the electric fields shown in Fig. 3(b).

**Figure 5:**
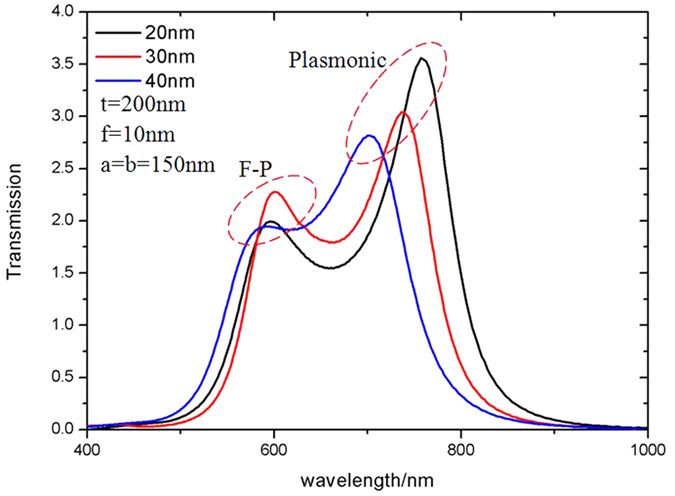
Transmission efficiency through *a* = *b* = 150 nm bowtie apertures defined in *t* = 200 nm thick silver film with different gap distances, black line: *g* = 20 nm, *f* = 10 nm; red line: *g* = 30 nm, *f* = 10 nm; blue line: *g* = 40 nm, *f* = 10 nm.

Figure 5 shows the effect of changing the gap, *g*, on the transmission spectra. The plasmonic resonance is red shifted as the gap distance increases while the resonant wavelength of the F-P resonance remains is less sensitive. This agrees with the hybridized model of the resonant effects in the bowtie aperture. Due to the changing dipole charge distribution, the plasmonic resonances are sensitive to the gap distance. However, the F-P resonance is principally determined by the overall length of the cavity and less sensitive to small gap differences.

**Figure 6:**
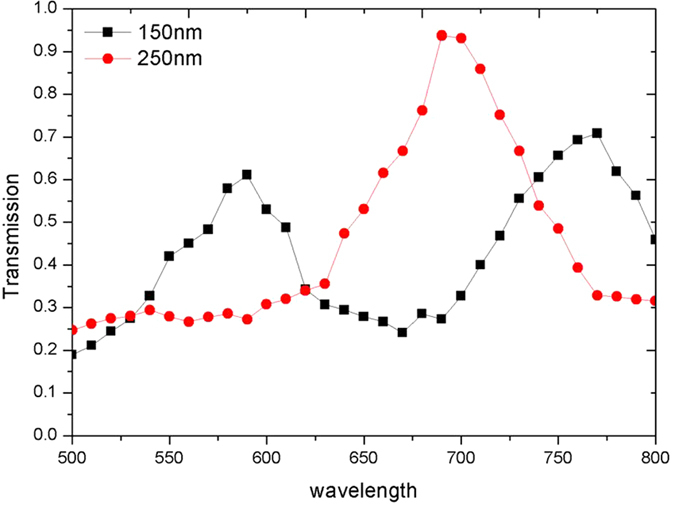
Measured transmission spectra for bowtie apertures with two outline dimension, *a* = *b* = 150 nm and *a* = *b* = 150 nm 250 nm defined in 200 nm thick silver film. Both apertures have a gap, *g* = 25 nm.

To verify the numerical results, the transmission spectra for *a* = *b* = 150 nm and *a* = *b* = 250 nm bowtie apertures are experimentally measured and shown in Fig. 6. Minor alignment variations between the two specimens prevent a direct comparison of the transmission amplitudes between the two test samples. The measured resonant wavelengths for the *a* = *b* = 150 nm aperture are λ_FP_ = 590 nm and λ_P_ = 770 nm. This agrees with simulation results which predicts λ_FP_ = 580 nm and λ_P_ = 713 nm. For the *a* = *b* = 250 nm aperture, the resonant plasmonic mode is outside the measurement range. The F-P resonance is measured at *λ* = 690 nm which agrees with the numerical result at 670 nm. These deviations can be attributed to minor deviations between the simulated and fabricated geometry as well as variations in the optical properties of the silver and silica.

In summary, the spectral resonances in nanoscale bowtie apertures was studied. Results demonstrate that the bowtie aperture has hybrid resonance characteristics: F-P resonance and plasmonic resonance. The F-P resonance is dominated by the film thickness while the plasmonic resonance is sensitive to the gap distance. Both resonances are closely related to the outline dimension of the aperture. These results reveal the physics behind the resonance effects and provide effective tuning methods to optimize bowtie apertures. To verify the numerical results, the transmission spectra of two bowtie apertures are experimentally measured. The experimental data is in excellent agreement with the numerical results. Understanding these effects provides significant insight to design the geometry of bowtie aperture for many applications benefiting from high optical resolution and near-field enhancement.

## Methods

### Simulations

Commercial finite different time domain software FDTD Solutions (Lumerical) is used to compute optical near field light transmission through the sub-wavelength bowtie aperture. A normally incident Gaussian beam of width *w* = 2 μm is used to excite the apertures from the substrate side. This illumination can be assumed to be well polarized and monochromatic. Using a Gaussian beam instead of a plane wave reduces non-physical reflections from radiation boundaries at glancing incidence. The transmission efficiency is calculated by normalizing the power passing through the aperture on the exit side to the power incident on the open area of the aperture. Silver is selected for its low losses at visible frequencies and represented by a Johnson and Christy^25^ model from the software database. The substrate is modeled with a refractive index *n* = 1.5 to represent quartz.

### Sample fabrication

**Figure 7:**
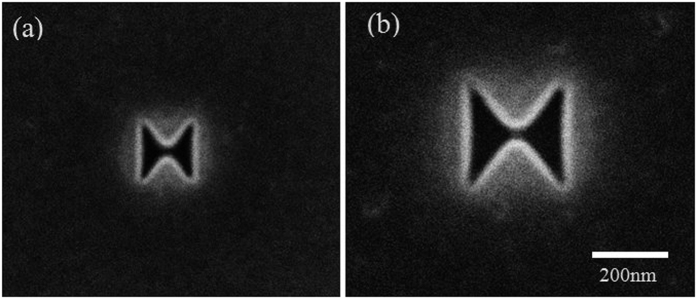
SEM pictures of FIB milled bowtie apertures in silver films with outline dimensions of (**a**) *a* = *b* = 150 nm and (**b**) *a* = *b* = 250 nm.

A 200 nm thick silver film is deposited on a fused quartz wafer by e-beam evaporated. The apertures are then Focused Ion Beam (FIB) milled using Ga+ ions using a FEI Helios Nanolab650 in the silver film. Figure 7 shows Scanning Electron Microscope (SEM) pictures of the apertures after milling.

### Measurement setup

**Figure 8:**
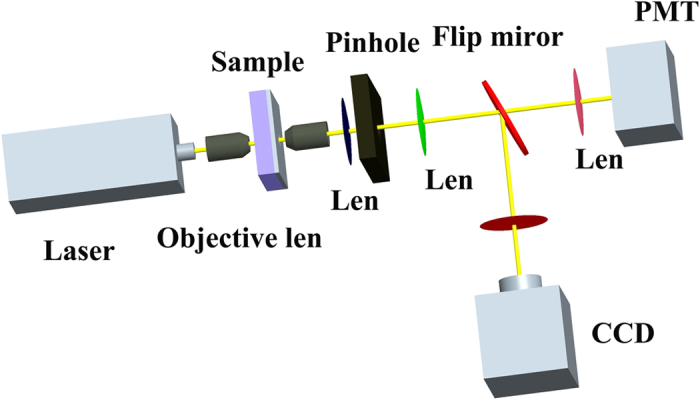
Schematic diagram of measurement setup.

Figure 8 illustrates the schematic experimental setup. A tunable laser output from an Optical Parametric Amplifier (OPA) pumped by an amplified ultrafast laser system is used as the light source. 50x microscope objectives lenses (N.A. = 0.7) are used to focus the laser beam onto the bowtie aperture and to collect transmitted light. A 50 μm pinhole is used to spatially filter transmitted light and helps to eliminate light other than what is transmitted by the aperture under study. The flip mirror allows the transmitted light to either be imaged by a CCD camera or focused onto a Photo Multiplier Tube (PMT) for measurement. During the experiment, the bowtie aperture is positioned in the laser focus. The transmission efficiency of bowtie apertures in different wavelengths is determined by measuring the PMT photon counts after a spectral calibration. During the experiment the wavelength of the incident laser beam is swept from 500 nm to 800 nm. This spectra measurement range is limited by our experimental conditions.

## Additional Information

**How to cite this article**: Ding, L. *et al*. Resonant Effects in Nanoscale Bowtie Apertures. *Sci. Rep.* **6**, 27254; doi: 10.1038/srep27254 (2016).
